# Exploratory biomarker analysis for treatment response in KRAS wild type metastatic colorectal cancer patients who received cetuximab plus irinotecan

**DOI:** 10.1186/s12885-015-1759-y

**Published:** 2015-10-20

**Authors:** Seung Tae Kim, Tae Jin Ahn, Eunjin Lee, In-Gu Do, Su Jin Lee, Se Hoon Park, Joon Oh Park, Young Suk Park, Ho Yeong Lim, Won Ki Kang, Suk Hyeong Kim, Jeeyun Lee, Hee Cheol Kim

**Affiliations:** 1 Division of Hematology-Oncology, Department of Medicine, Samsung Medical Center, Sungkyunkwan University School of Medicine, Seoul, Korea; 2 Department of Pathology & Translational Genomics, Samsung Medical Center, Sungkyunkwan University School of Medicine, Seoul, Korea; 3 Samsung Genome Institute, Samsung Biological Research Institute, Seoul, Korea; 4 Department of Surgery, Samsung Medical Center, Sungkyunkwan University School of Medicine, Seoul, Korea; 5 Colorectal Cancer Center, Samsung Medical Center, Sungkyunkwan University School of Medicine, Seoul, Korea; 6 Innovative Cancer Medicine Institute, Samsung Medical Center, Seoul, Korea

**Keywords:** KRAS, Cetuximab, Colorectal cancer

## Abstract

**Background:**

More than half of the patients selected based on *KRAS* mutation status fail to respond to the treatment with cetuximab in metastatic colorectal cancer (mCRC). We designed a study to identify additional biomarkers that could act as indicators for cetuximab treatment in mCRC.

**Methods:**

We investigated 58 tumor samples from wild type *KRAS* CRC patients treated with cetuximab plus irinotecan (CI). We conducted the genotyping for mutations in either *BRAF* or *PIK3CA* and profiled comprehensively the expression of 522 kinase genes.

**Results:**

*BRAF* mutation was detected in 5.1 % (3/58) of patients. All 50 patients showed wild type PIK3CA. Gene expression patterns that categorized patients with or without the disease control to CI were compared by supervised classification analysis. *PSKH1, TLK2* and *PHKG2* were overexpressed significantly in patients with the disease control to IC. The higher expression value of *PSKH1* (r = 0.462, p < 0.001) and *TLK2* (r = 0.361, p = 0.005) had the significant correlation to prolonged PFS.

**Conclusion:**

The result of this work demonstrated that expression nature of kinase genes such as PSKH1, TLK2 and PHKG2 may be informative to predict the efficacy of CI in wild type KRAS CRC. Mutations in either BRAF or PIK3CA were rare subsets in wild type KRAS CRC.

## Background

Colorectal cancer (CRC) has been a significant cause of morbidity and mortality throughout the world [1]. Metastatic CRC (mCRC) is associated with a particularly poor prognosis. Despite progress in cytotoxic chemotherapy during past decade, the five year survival rate for mCRC remains below 10 % [2]. There have been recent and rapid advances in the development of agents targeted against components of receptor tyrosine kinase signaling cascades for use in cancer-therapy. A monoclonal antibody (mAb) against epidermal growth factor receptor (EGFR), referred to as cetuximab, is a product of this movement, and has been implemented to treat selected mCRC patients.

However, only 10–20 % of the selected mCRC patients benefit from anti-EGFR therapy [3–6], highlighting a distinct need for individualized treatment. Following the discovery that mutations in *KRAS* are associated with resistance to anti-EGFR treatment, determination of *KRAS* status is now recommended in mCRC patients before starting anti-EGFR therapies. Despite the application of these selective strategies, less than half of the chosen wild type KRAS patient population benefits from anti-EGFR treatment [7–9]. More recently, other oncogenic alterations such as mutations in *BRAF* and *PIK3CA* were identified as candidates associated with resistance to anti-EGFR therapies in wild type KRAS patients [8, 10–13]. However, there is still a need to identify and confirm additional biomarkers that can be used to more accurately select wild type mCRC patients that will respond to anti-EGFR therapy.

Protein kinases control many cellular processes including metabolism, transcription, cell cycle progression, cytoskeletal rearrangement, cell movement, apoptosis, and differentiation [14, 15]. Therefore, protein kinases are essential targets for molecular therapy. Indeed, various protein kinase inhibitors have been shown to be effective against cancer cells. Cancers often result from the interconnectivity of complex pathways, some of which are not well understood. For this reason, we surmise that the anti-tumor activity of cetuximab may be affected by various kinase genes involved different pathways.

In order to identify additional selective biomarkers for CI indication, we genotyped wild type KRAS colorectal tumor samples from patients that received CI treatment for mutations in either *BRAF* or *PIK3CA*, and profiled comprehensively the expression of 522 kinase genes involved different pathways.

## Methods

### Patients and samples

Fifty-eight mCRC patients with CI, for whom collection of tumor samples was part of their routine care and treatment, were enrolled for this analysis. Patients were treated by CI after confirming *KRAS* mutational status (wild type). The tumor samples were sufficient to study additional biomarkers, such as genotyping for *BRAF* and *PIK3CA* and targeted gene expression profiling. In all cases, we reviewed patient age at diagnosis, gender, Eastern Cooperative Oncology Group (ECOG) performance status, the number of involved organs, metastatic site, and chemotherapy data. All hematoxylin and eosin stained slides were reviewed, and representative paraffin blocks were selected for further studies.

### DNA extraction and mutation analysis for BRAF and PIK3CA

DNA was extracted from five 10-μm thick formalin fixed paraffin embedded (FFPE) sections containing a representative portion of each tumor block, using the QIAamp DNA Mini kit (Qiagen, Hilden, Germany). A pathologist reviewed each slide and verified the presence of adequate tumor tissue with greater than 50 % representative malignant cells.

Peptide nucleic acid-locked nucleic acid (PNA–LNA) PCR clamp reactions were carried out using the PNA-Clamp™ *BRAF*, and PNA-Clamp™ *PIK3CA* Mutation Detection Kits (Panagene, Inc., Daejeon, Korea), as described previously. Briefly, this reaction consists of the following; all reactions were done in 20 μl volumes using 10–25 ng template DNA, primer and PNA probe set, and SYBR Green PCR master mix. All necessary reagents are included with the kit. Real-time PCR reactions of PNA-mediated clamping PCR were performed using a CFX 96 system (Bio-Rad, USA). PCR conditions started with a 5 min hold at 94 °C, followed by 40 cycles of 94 °C for 30 s, 70 °C for 20 s, 63 °C for 30 s, and 72 °C for 30 s. Detection of each of mutation in *BRAF* exon 15, and 3 mutations in *PIK3CA* exons 2 & 9 was possible using one-step PNA-mediated real-time PCR clamping.

### Targeted gene expression profiling

The Nanostring-based multigene assay was performed in tissue samples of 58 patients who received cetuximab-based therapy for mCRC. Total RNA was extracted from one or two sections of 4-μm thick FFPE tumor sections using the High Pure RNA Paraffin kit (Roche Diagnostic, Mannheim, Germany) after removing non-tumor elements by manual macrodissection guided by hematoxylin and eosin stained slides. nCounter® GX Human Kinase Kit (NanoString Technologies, Seattle, WA, USA) was used for gene expression analyses. One hundred nanogram of total RNA was hybridized with the pre-built code set of 522 genes for 18 h at 65 °C and processed according to manufacturer’s instruction [16].

### Ethics statement

The institutional review board of the Samsung Medical Center (SMC) approved the process of acquiring tissue samples in mCRC patients with cetuximab-based therapy. All study participants provided a written informed consent form, which was approved by the institutional review board. The methods in this study were carried out in accordance with the approved guidelines by SMC and all experimental protocols were approved by the ethics committees of SMC.

### Statistical analysis

To discover differential expression of 522 kinase genes between two groups (partial response plus stable disease vs. progressive disease), t-test is applied after the data is normalized by quantile method. Response definitions that differentiated two groups were according to Response Evaluation Criteria in Solid Tumors (RECIST) 1.1. To identify gene set that is associated with disease control, t-test statistics of 522 genes were further assessed by Pathway Activity inference using Condition-responsive genes (PAC). Spearman’s test was used to assess the correlation between progression free survival (PFS) and expression vales of selected kinase genes that related to the disease control for cetuximab. Results were considered statistically significant when p-values were < 0.05. Gene expression difference analysis and pathway analysis were performed using R (version 2.15 and package 2.10) (http://www.R-project.org), and other statistical analyses were performed using SPSS software (SPSS Inc., Chicago, IL, USA).

### Pathway association analysis

To identify gene set that associated with disease control (partial response plus stable disease vs. progressive disease), we conducted Efron-Tibshirani’s gene set analysis (GSA) maxmean test embedded in the BRB Array Tool. We examined 92 gene sets in Biocarta Pathway database and the threshold of determining significant gene sets is 0.01.

## Results

### Clinicopathological features

As summarized in Table 1, 58 wild type KRAS mCRC patients of a median age of 59 years (range, 39–91) received CI treatment. The study included 33 (56.9 %) male and 25 (43.1 %) female patients. Most of the patients (96.6 %) had a good performance status (ECOG 1) and over half of the patients (75.9 %) had metastatic disease at diagnosis. More than half of patients (51.7 %) had three or more metastatic lesions and 91.4 % received CI as third or more line treatment. Only 18 patients (31 %) had prior anti-VEGF therapy before CI.

**Table 1:** Baseline characteristics of the patients enrolled in this study

Characteristics	Number of patients N (%)
Median age (years, range)	59 (39–91)
Sex	
Men	33 (56.9)
Female	25 (43.1)
ECOG performance status	
1	56 (96.6)
2	2 (3.4)
Disease status	
Recurrent	14 (24.1)
Metastatic	44 (75.9)
Number of metastatic sites	
≤2	25 (43.1)
2<	33 (56.9)
Number of prior regimen for advanced disease	
1	5 (8.6)
2	46 (79.3)
3	6 (10.3)
4	1 (1.7)
Previous anti-VEGF treatment	
Bevacizumab	18 (31.0)
BRAF status (V600E)	
Mutant	3 (5.1)
Wild type	51 (88.1)
Unknown	4 (6.8)
PIK3CA status	
Mutant	0 (0.0)
Wildtype	50 (86.2)
Unknown	8 (13.8)

### Frequency of BRAF and PIK3CA mutations

*BRAF* mutation status was assessed in 54 of 58 (93.1 %) patients. Three of these (5.6 %) patients harbored *BRAF* mutations (V600E). *PIK3CA* mutation status was evaluated in 50 of 58 (86.2 %) patients, and all 50 patients showed wild type PIK3CA (Table 1). CI did not reveal the anti-tumor effect in all 3 patients with BRAF mutation.

### Expression of kinase genes and cetuximab treatment outcome

**Fig. 1:**
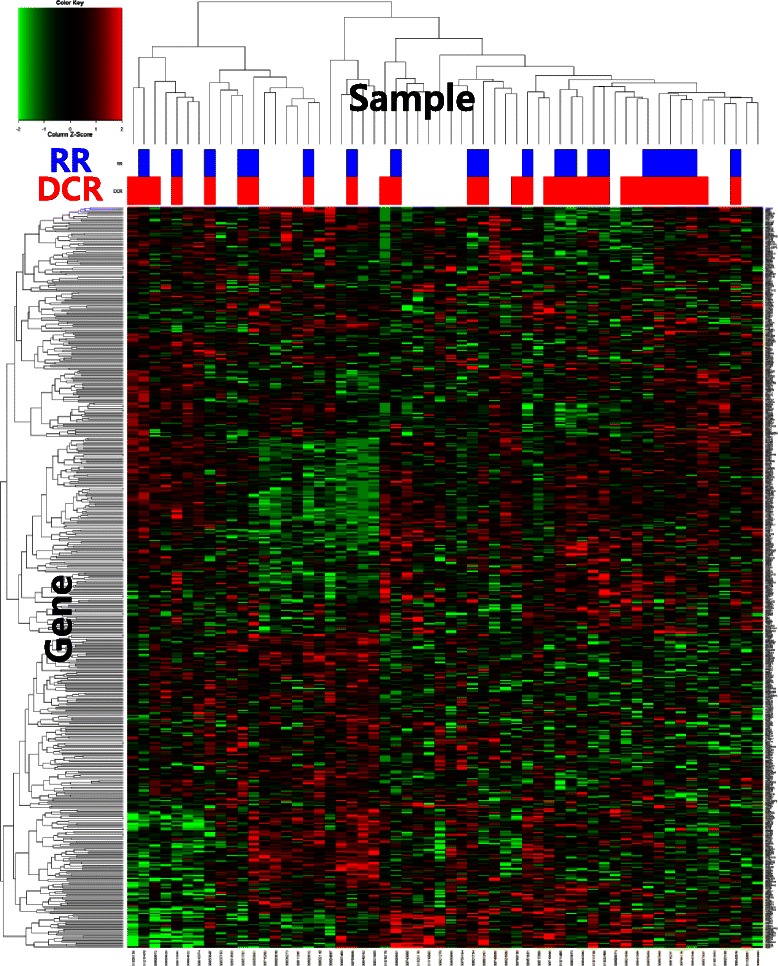
Supervised clustering of 522 kinase genes that are differentially expressed between patients with and without tumor response to CI and between patients with and without the disease control to CI. RR; complete response plus partial response: DCR; RR plus stable disease

**Fig. 2:**
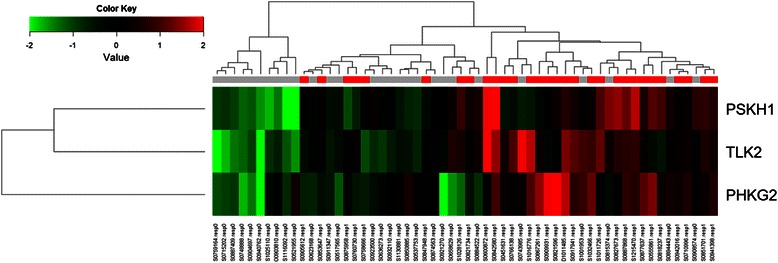
Kinase genes (PSKH1, TLK2 and PHKG2) that were differentially expressed between patients with and without the disease control to CI

Among the 58 patients receiving cetuximab-based therapy, 21 achieved a confirmed partial response and nine had stable disease, resulting in an overall response rate (ORR) of 36.2 % (21/58) and disease control rate (DCR) 51.7 % (30/58) (Table 2). There were 522 kinase genes analyzed by nCounter assay and a supervised method was used to find statistically significant differentially expressed genes between patients with and without the disease control for CI. A hierarchical clustering analysis was then performed on the samples based on expression value of 522 genes in 58 patients (Fig. 1). We found three kinase genes such as PSKH1, TLK2 and PHKG2 that were presented significantly higher expression levels in patients with the disease control to CI (Fig. 2).

**Table 2:** Treatment outcomes of cetuximab therapy and the corresponding number of patients

Treatment outcomes	Number of patients N (%)
Response	
Complete response	0 (0.0)
Partial response	21 (36.2)
Stable disease	9 (15.5)
Progressive disease	28 (48.3)
Response rate	21 (36.2)
Disease control rate	30 (51.7)

### Progression free survival and expression of PSKH1, TLK2 and PHKG2

**Fig. 3:**
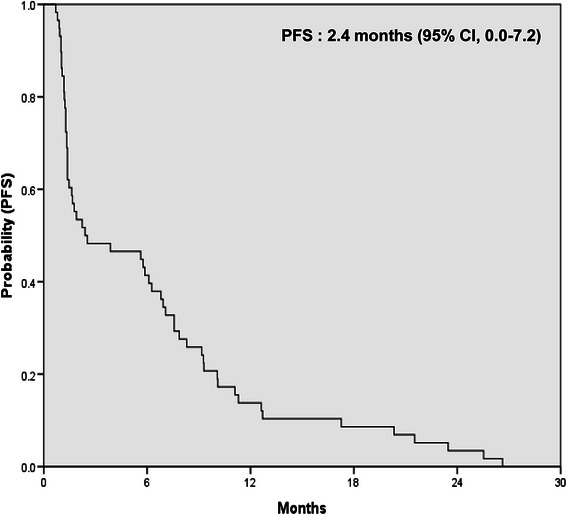
Kaplan-Meier estimate of progression free survival (PFS)

**Fig. 4:**
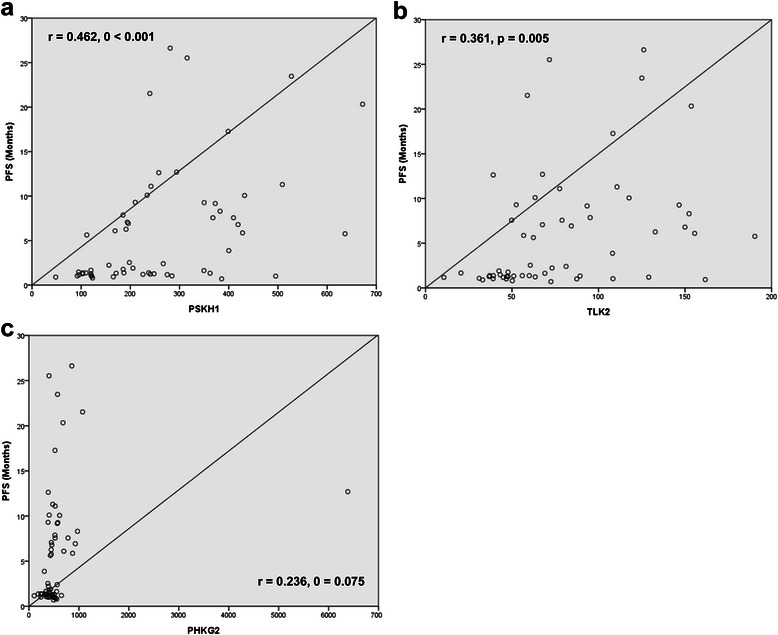
The correlation between PFS and the expression value of (a) PSKH1, (b) TLK2 and (c) PHKG2

All 58 patients with cetuximab-based therapy were analyzed for progression free survival (PFS) and exhibited a median PFS of 2.4 months (95 % CI, 0.0–7.2) (Fig. 3). The correlation was analyzed between PFS and the expression values of PSKH1, TLK2 and PHKG2. The higher expression value of PSKH1 (r = 0.462, p < 0.001) (Fig. 4a) and TLK2 (r = 0.361, p = 0.005) (Fig. 4b) had the significant correlation to prolonged PFS. However, the statistically significant correlation was not observed between PHK2G2 and PFS (r = 0.236, p = 0.075) (Fig. 4c).

### Signaling pathways related to the anti-tumor activity of CI

We performed pathway analysis using gene set enrichment analysis for expression of 522 kinase genes. For the disease-controlled cohort, 3 out of 92 investigated Biocarta pathway gene sets passed the 0.01 significance threshold as verified by Efron-Tibshirani’s GSA maxmean test. These included the CCR3 signaling pathway, the cdMac signaling pathway, and the integrin signaling pathway (Table 3).

**Table 3:** Results of the gene-set enrichment analysis of the Biocarta pathway for disease control

	Biocarta Pathway	Pathway description	Number of genes	Efron-Tibshirani’s GSA test p-value
1	h_CCR3Pathway	CCR3 signaling in eosinophils	8	0.005 (−)
2	h_cdMacPathway	Cadmium induces DNA synthesis and proliferation in macrophages	6	0.005 (−)
3	h_integrinPathway	Integrin Signaling Pathway	10	0.005 (−)

## Discussion

Despite exclusion of 30–40 % of patients with *KRAS* mutant tumors, cetuximab-based therapy fail in more than half of CRC patients harboring wild type KRAS [4, 9, 17–19]. Recently, mutations in other downstream effectors of the EGFR pathway, such as *BRAF, NRAS*, and *PIK3CA* have also been implicated as possible predictors for anti-EGFR response [12, 18, 20, 21]. Overall, the benefit of analyzing these candidates has been difficult to determine due to low mutation frequencies for individual markers. In an attempt to identify additional biomarkers for the efficacy of cetuximab-based therapy in CRC patients with wild type KRAS, we performed targeted gene expression profiling for 522 kinase genes in addition to genotyping for BRAF and PIK3CA. Our analysis suggested that the overexpression of PSKH1, TLK2 and PHKG2 may be a considered a potential biomarker to predict the efficacy of CI in wild type KRAS CRC. Moreover, the higher expression value of PSKH1 (r = 0.462, p < 0.001) and TLK2 (r = 0.361, p = 0.005) had the significant correlation to prolonged PFS. However, among 522 kinase genes, any gene didn’t have significant prognostic value for overall survival (OS). We also conducted the multivariate analysis (Cox proportional hazard modeling) for PFS with clinical variable and expression nature of kinase genes such as PSKH1, TLK2 and PHKG2. Univariate analysis showed that prolonged PFS was significantly associated with metastatic sites of ≤ 2, and expressed of PSKH1, TLK2 and PHKG2. In multivariate analysis, only expressed of PSKH1 (hazard ratio [HR], 2.219; 95 % confidence interval [CI], 1.085 TO 4.541; p = 0.029) was significantly with prolonged PFS.

We used an nCounter assay to analyze the expression of kinase genes. The nCounter assay can profile 522 human kinase genes in one reaction tube with only 100 ng of total RNA. Previously, we compared the results of an nCounter assay with immunohistochemistry results using three biomarkers (EGFR, HER2 and MET) in a gastric cancer (GC) model [22]. Relative to immunohistochemistry findings, the NanoString-based assay sensitivities and specificities were 85.7 % and 82.8 % for EGFR, 100 % and 97.2 % for HER2, and 100 % and 100 % for MET, respectively. Hence, nCounter assays may be feasible as a relatively reliable option in the clinic, where only small amount of RNA (~100 ng) can be rendered from paraffin-embedded tissues. Based on our previous study, NanoString-based kinase assay has been used as a feasible screening tool for initially identifying patients who have GC with overexpressed drug targets in our institution. This study suggested that nCounter assays could be used efficiently as a platform for implementing personalized medicine of CRC.

*PSKH1* is a 424 amino acids long ubiquitously expressed autophosphorylating humane protein serine kinase [23, 24]. PSKH1 has been known to have a useful structural and regulatory role in the maintenance of the Golgi apparatus [25]. TLK2 is a nuclear serine/threonine kinase which is a cell cycle check point regulating chromatin assembly and is inactivated in response to DNA damage [26]. Although the higher expression value for PSKH1 and TLK2 is likely to be related to prolonged PFS as well as antitumor effect for CI in CRC patients in this analysis, the molecular determinants for overexpression for PSKH1 and TLK2 are unknown for the effect of cetuximab. Further functional analysis of these kinase genes is needed to apply these markers in clinical practice.

Assessment of some mutations could improve the objective response rate; objective response rates were 24.4 % in unselected population, 36.3 % in population harboring the wild type KRAS, and 41.2 % in the *KRAS*, *BRAF*, *NRAS*, and *PIK3CA* exon 20 wild type populations [18]. However, cetuximab still fails to meet expectations in more than half of patients who were selected via genotyping of multiple genes. There are some limitations in our study. A small sample size, heterogeneous patient population, retrospective nature, and limited validated data didn’t allow us to draw any significant conclusions. Especially, our study included only 3 patients with BRAF mutation. Thus, the role of BRAF mutation as predictive marker for cetuximab needs to be validated and studied. Tissue availability was also a potential limitation of the current retrospective biomarker-analysis. It is better to verify the PSKH1, TLK2, and PHKG2 expression identified by nCounter assay in tissues using independent methods such as in situ hybridization, immune histochemistry or RT-qPCR. However, patients analyzed in this study had refractory cancer. Among these patients, it was difficult to get the tumor tissue for biomarker-analysis. Nevertheless, our attempts to whether expression of kinase genes could serve as biomarkers for the efficacy of cetuximab therapy in wild type KRAS CRC patients is worth noting.

Many crucial signaling pathways become dysregulated during cancer initiation and progression. Identifying the pathways involved and quantifying their deregulation is an important step toward understanding carcinogenesis [27–30]. Because advanced therapies target specific pathways, pathway-level understanding is also a necessity in the development of personalized cancer treatments. Previously, we found that *KRAS* mutant CRC patients with low RAS signature scores were likely to benefit from treatment with cetuximab, irinotecan plus simvastatin [31]. This finding suggested that differential RAS signaling in *KRAS* mutant CRC patients is a useful biomarker for choosing the proper treatment regimen. Based on our analysis, CCR3, cdMac, and integrin pathways are significantly associated with the activity of cetuximab as the down-regulation of these pathways correlated with an observed increase in cetuximab efficacy, though these results must be validated. Additional, work will also need to be done to evaluate whether the CCR3, cdMac, and integrin pathways are interconnected with the EGFR signaling that was known as the key pathway for the effect of cetuximab. Many new investigational agents, such as inhibitors of MAP2K, PI3K, mTOR, EGFR, and BRAF will be extremely useful for elucidating these networks [32]. These pathway-based approaches will be used as novel algorithms for precision medicine.

In conclusion, our study suggested that expression nature of kinase genes such as PSKH1, TLK2 and PHKG2 may be informative to predict the efficacy of CI in wild type KRAS CRC. In addition to known EGFR pathway, the CCR3, cdMac, and integrin pathways may also be useful in predicting cetuximab response in these patients. Our findings warrant further independent validation. In the future, multiple-biomarker assessment will be needed in mCRC patients in order to optimize identification of patients that will benefit from cetuximab.

## Conclusion

The result of this work demonstrated that CI did not have anti-tumor activity in BRAF mutant CRC. Additionally, expression nature of kinase genes such as PSKH1, TLK2 and PHKG2 may be informative to predict the efficacy of CI in wild type KRAS CRC.

### Ethics statement

The Ethics Committee at Samsung Medical Center approved the study in accordance with the Declaration of Helsinki. All individuals gave written informed consent for participation in the study.
